# A Prospective Clinical Trial of Prolonged Fasting in Healthy Young Males and Females—Effect on Fatigue, Sleepiness, Mood and Body Composition

**DOI:** 10.3390/nu12082281

**Published:** 2020-07-30

**Authors:** Boya Nugraha, Amin Riat, Samaneh Khoshandam Ghashang, Luqman Eljurnazi, Christoph Gutenbrunner

**Affiliations:** Department of Rehabilitation Medicine, Hannover Medical School, 30625 Hannover, Germany; aminhajamer@gmail.com (A.R.); samaneh.khoshandam@googlemail.com (S.K.G.); luqman_eljurnazi@yahoo.com (L.E.); gutenbrunner.christoph@mh-hannover.de (C.G.)

**Keywords:** fasting, Ramadan, health, fatigue, mood, body composition

## Abstract

Fasting during a long period (17–18 h/day) may affect daily performance. Fatigue is one of important parameters to observe for this effect. This study aimed to determine the effect of Ramadan fasting (RF)—particularly on fatigue in both young males and females. Sleepiness, mood-related symptoms (MRSs) and body composition (BC) were determined, too. Thirty-four young males and females were recruited and performed RF. They were assessed for fatigue, sleepiness, MRS and BC at T1 (one week before RF), T2 (mid of RF), T3 (last days of RF), T4 (one week after RF) and T5 (one month after RF). The assessments were done in the morning, except for fatigue and sleepiness, which were also assessed in the afternoon and evening of T1 to T5. This study observed numerous positive effects to RF. After they began fasting and compared to T1, participants experienced (1) significantly less fatigue; (2) small to large improvement of MRSs; and (3) positive changes relating to BC. RF did not appear to have a significant effect on participants’ sleepiness scores. Ultimately, this study illustrates how prolonged fasting, like RF, benefits the youth, particularly by decreasing fatigue, improving MRSs and positively impacting BC.

## 1. Introduction

Fasting has been practiced for centuries by billions of people worldwide. The benefits of different types of fasting have also been recorded. Intermittent fasting (IF) is a period of voluntary abstinence from food and drink without focusing on the type of food and drinks are consumed [1]. Studies show that IF is beneficial for weight loss [2], IF also reduces fat mass, but not skeletal mass in old females [3]. Studies also illustrate that IF reduces weight loss and insulin requirement in diabetic patients (both type 1 and type 2) [4], reduces inflammatory markers in cardiovascular diseases [5], results in improved peripheral and central blood pressure control in hypertensive patients with and without chronic kidney diseases [6] and reduces toxicity and facilitate effective chemotherapy in cancer patients [7]. IF could benefit against a different type of diseases related to aging [8,9] and it has many other health benefits [9,10]. IF has also become a lifestyle in combination with exercise [11].

Ramadan fasting (RF) is one type of IF. The difference between RF and other types of IFs is that it is only performed during the month of Ramadan (a month the Islamic calendar year) and people engaged in RF must abstain from food, drink, sex and smoke from dawn to dusk. There are exceptions including for people who are sick, women during the menstrual periods and those who are traveling. Though RF is generally obligatory for Muslims, many studies have reported the benefits of RF. Studies have analyzed the effect of RF in patients with various conditions, such as diabetes [12,13], kidney-related problems [14] and asthma [15]. In patients, RF should be carefully monitored by health professionals. Other studies also reported the effect of RF on young males [16,17,18], workers [19], tennis players [20] and taekwondo athletes [21].

A long period of fasting could influence different aspects of the human body, including at genetic level [22,23] and its phenotypic manifestations; for example, one study showed the changing in health-related quality of life and body composition (BC) parameters [16]. Another study showed the alteration of creatinine levels during a long fasting period in young males, although it is still in health range [17].

Fatigue is one of the important factors to be determined for daily performance [24], as it is strongly related to poor physical performance [25], including during RF. Fatigue is defined as a subjective experience and includes such symptoms as rapid inanition, persisting lack of energy, exhaustion, physical and mental tiredness and apathy [26]. Therefore, fatigue could be one of the important factors to be determined during RF, as it could also influence daily performance, either at the office, school, university or any other activities.

Interesting results were also reported in a recent study that demonstrated the beneficial effect of fasting in the young male in relation to fatigue and health-related quality of life [16]. It was conducted during summertime in Germany which had a long fasting period (18–19 h/day). However, it had some limitations, including that it was performed only in young males and it was only assessed in the morning time. Meanwhile, the level of fatigue could be different between morning, afternoon and evening. It would be also of interest to determine the effect of RF in both sexes. Therefore, the main objective of this study was to determine the level of fatigue during RF, in both genders.

## 2. Materials and Methods

The local ethics committee of Hannover Medical School approved this study (Ethics No. 7242; Registration code of the trial: DRKS00017640). This study was in line with the Declaration of Helsinki. It was performed from May to July 2017. The RF itself was performed for 30 days (26 May–24 June 2017). Study center was Department of Rehabilitation Medicine, Hannover Medical School, Hannover, Germany. This study was evaluated at five different assessment time points (T1, T2, T3, T4 and T5) and in the morning, afternoon and evening (see Table 1).

### 2.1. Participants

Thirty-four healthy participants (male and female) were recruited. All participants were healthy (free from pain and psychiatric disorders). Most of them were students at Hannover Medical School. They were older than 18-year-old and planned to fast the whole month of Ramadan. All participants comprehended the English or German language. Participants were excluded if they broke the fast for more than 7 days. All participants participated in this study after signing informed consent.

### 2.2. Endpoints

Fatigue was the primary endpoint and assessed by using the fatigue severity scale (FSS). It is a self-administered questionnaire with nine questions. Every question has statements that are scored from 1 (strongly disagree) to 7 (strongly agree) [27]. Sleep problems were assessed by using Epworth sleepiness scale (ESS). ESS is also a self-reported questionnaire with eight item questions. Every question can be scored on a 4-point measuring tape (0–3). Final score of ESS is the sum of 8 questionnaires which can range from 0 to 24. The higher the ESS score, the higher that person’s average propensity in daily life to daytime sleepiness [28]. FSS and ESS questionnaires were taken in the morning (between 07:00 and 10:00), afternoon (between 13:00 and 15:00) and evening (between 19:00 and 20:00). In the morning at all assessment time points (T1–T5), participants filled in ESS and FSS at study center. In the afternoon and evening, they did it outside study center (e.g., at university or home).

Mood-related symptoms (MRSs) were determined by using Beck’s depression inventory (BDI)-II and hospital and anxiety depression scale (HADS). BDI-II is a self-administered questionnaire to measure the severity of depression with 21 questions. The total score of BDI-II ranges from zero to sixty-three. The higher the BDI-II score is, the more severe the depression [29]. HADS is also a self-reported questionnaire that consists of fourteen questions. Seven items are related to anxiety, and seven items are related to depression [26]. A score of 8 or more reveals anxiety or depression symptoms. BDI-II and HADS were determined in the morning at all assessment time points (T1-T5) at study center.

BC parameters were measured by using InBody 230 (Model MW160, InBody Co., Ltd, Seoul, Korea). The latter measurements include body fat percentage (BFP), body weight (BW), body water mass (BWM), skeletal muscle mass (SMM), body fat mass (BFM) and fat-free mass (FFM). This machine was also used to estimate basal metabolic rate (BMR). Body mass index (BMI) was calculated by measuring the height of the participants manually (using measuring tape) and calculated by using following formula: BMI = mass/(height)^2^; where mass in kg; height in m. BC parameters were determined only in the morning at all assessment time points (T1 to T5) at study center.

### 2.3. Sample Size Calculation

Sample size was calculated based on previous study [16] that showed partial eta square for fatigue (FSS; η^2^ = 0.151; [16]) that resulted the effect size (ES) of (f) = 0.4217. The type I error probability was set by 0.05 with power (ß) of 0.95. It resulted to have at least 24 participants. Considering 25% of participants dropped out, the total sample size should be at least 32 participants.

### 2.4. Statistics Evaluation

The study objective aimed at assessing the level of fatigue before, during and after Ramadan in three different time points: morning, afternoon and evening. Depending on the distribution of the data, analysis of the primary endpoint was done by using either mixed-model ANOVA or Two-way Friedman ranked test. Secondary endpoints, like sleepiness, MRSs and BC parameters were also investigated. Depending on the distribution of the data, secondary endpoints were analyzed by using either ANOVA repeated measure or Two-way Friedman ranked test. The post hoc tests were performed, and significances were adjusted by using Bonferroni correction. Missing data were replaced by using mean imputation method. Kolmogorov–Smirnov test was used to determine the distribution of the data. Significance was set at *p* < 0.05. SPSS 26 (IBM, New York City, NY, USA) was used to analyze the data.

ES for FSS and ESS were calculated by using following formula: η^2^ = Z^2^/(N − 1).

ES for BC parameters and MRSs were calculated by using following formula: (µ_1_ − µ_2_)/SD; SD = standard deviation.

## 3. Results

### 3.1. Recruitment of the Participants

**Figure 1:**
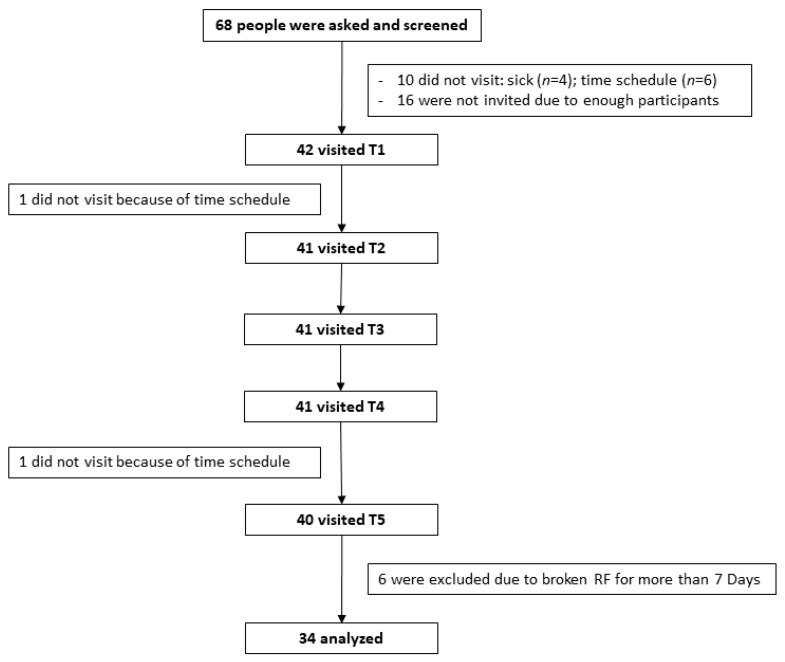
Flow chart recruitment of participants.

Sixty-eight people were asked to participate in this study. Fifty-two participants were invited, with 42 attending at T1. Due to several reasons, including sickness, time schedule and other reasons, only 34 participants remained as completers and is included in the analysis (Figure 1).

### 3.2. Baseline Characteristics of Participants

Table 2 shows the baseline characteristics of all participants. Additionally, statistics tests to compare male and female participants were performed. There were no significant differences in age and race between male and female participants. Fatigue, MRSs and sleepiness scores were not significantly different. As expected, almost all BC parameters between males and females showed significant differences, except BMI.

### 3.3. Fatigue and Sleepiness during RF

**Figure 2:**
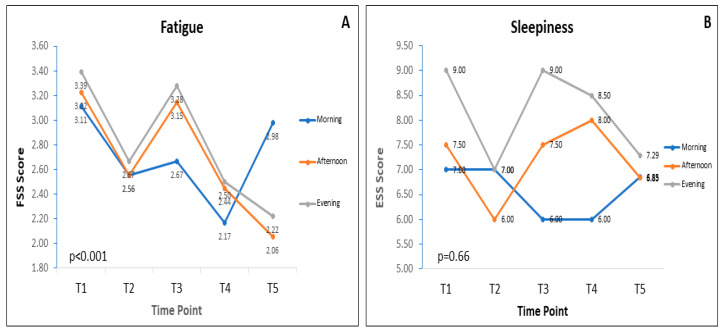
Patterns of fatigue (**A**) and sleepiness (**B**) scores during RF at T1 (one week before RF; *n* = 34), T2 (mid of RF; *n* = 34), T3 (last days of RF; *n* = 34), T4 (one week after RF; *n* = 34) and T5 (one month after RF; *n* = 34). Both fatigue and sleepiness were assessed in the morning, afternoon and evening. Data presented as median.

Figure 2 demonstrates the pattern of fatigue (2A) and sleepiness (2B) scores at all times of the study in the morning, afternoon and evening. Significant differences were observed in fatigue scores (*p* < 0.001; two-way Friedman rank test). The patterns of fatigue score, particularly in the morning tend to improve from baseline (T1) to T4 and turned back to the baseline level at T5. The pattern of alterations of fatigue in the afternoon and evening were similar (except at T3) and tend to decrease until T5.

There were no significant difference in sleepiness scores (*p* = 0.66; two-way Friedman rank test). The patterns of sleepiness scores in the afternoon and evening were similar.

Table 3 contains the data of each day-time group (morning, afternoon and evening). The significant differences were observed only in fatigue scores. Post hoc analysis showed that fatigue score was significantly different in the morning and evening. Specifically, significant differences in the morning were observed when comparing T1 and T4 (*p* < 0.05). In the evening, the significant differences were observed when comparing T1 and T5 (*p <* 0.01). No significant differences were observed in the sleepiness score.

**Figure 3:**
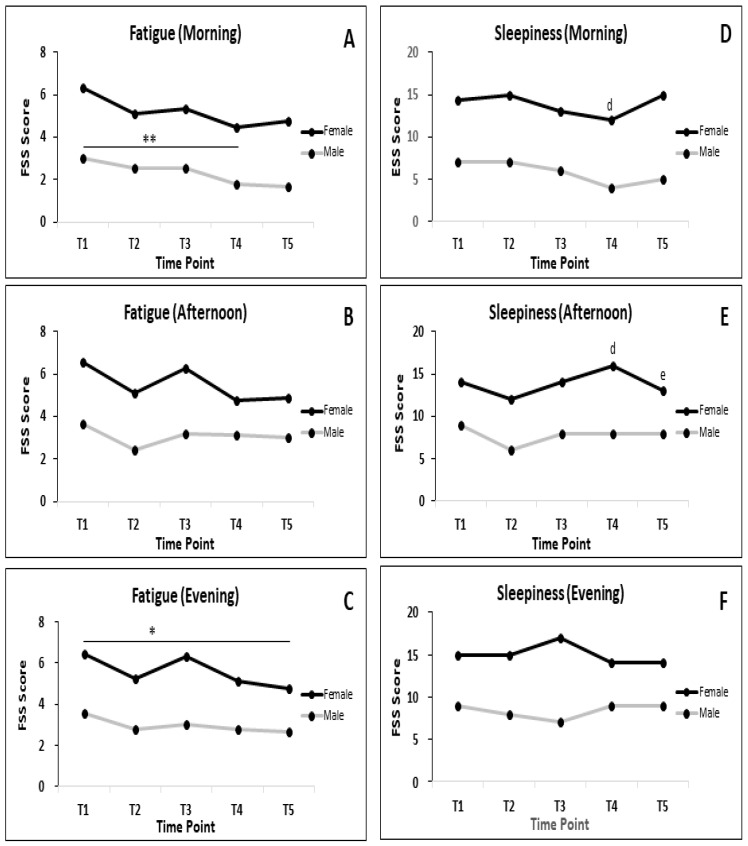
(**A**,**D**) Patterns of fatigue and sleepiness during RF in male and female in the morning; (**B**,**E**) afternoon and (**C**,**F**) and evening; (d,e) significant difference between male and female at T4 and T5, respectively. *n* at each time point for male = 19, female = 15. Data presented as median. * *p* < 0.05; ** *p* < 0.01.

The effect of RF on fatigue and sleepiness is shown in Figure 3. The significant differences concerning fatigue could only be observed in the male in the morning (T1 vs. T4; *p <* 0.01; Figure 3A); and female in the evening (T1 vs. T5; *p* < 0.05; Figure 3C). Although no significant differences of sleepiness score, the authors also separately analyzed the data comparing the score of fatigue and sleepiness of male and female. This also shows in Figure 3. The data clearly shows that females tend to have higher values in both FSS and ESS. The significant differences between males and females was only observed in ESS scores in the morning at T4 (Figure 3D) and in the afternoon at T4 and T5 (Figure 3E).

Table 4 demonstrates the ES of FSS and ESS between T1 and T3. The results show that the ES of FSS and ESS between T1 and T3 in the morning, afternoon and evening time in all participants, in only females and males was small.

### 3.4. MRSs during RF

**Figure 4:**
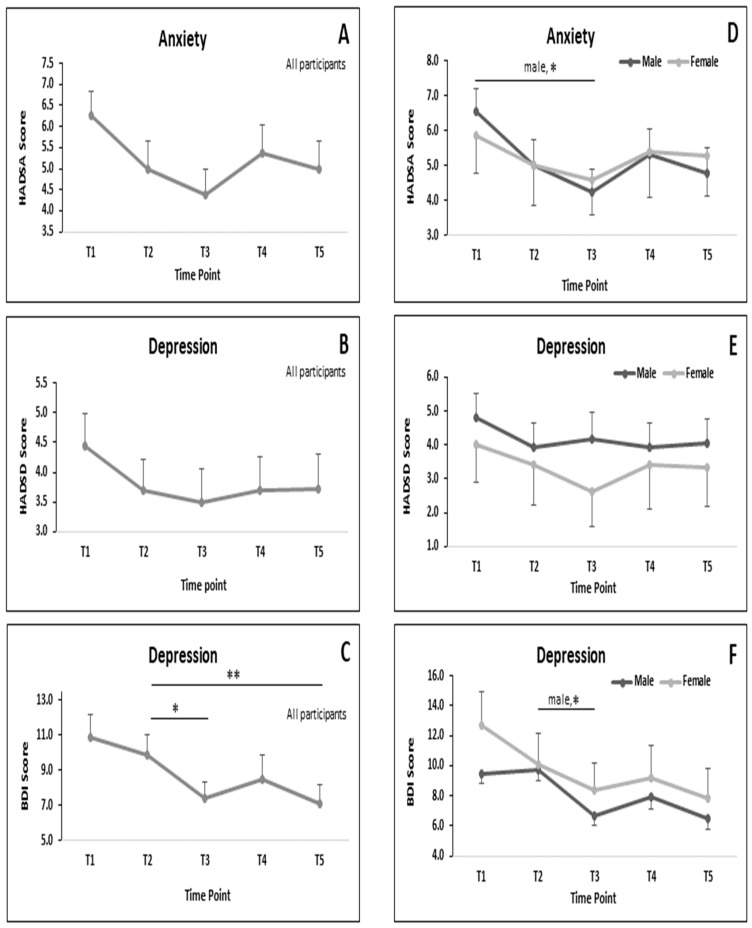
Patterns of MRSs during RF. (**A**–**C**) All participants; (**D**–**F**) subgroup male and female. Data presented in mean and SEM. * *p* < 0.05; ** *p* < 0.01.

Figure 4 demonstrates the effect of RF on MRSs. Significant differences of depression score were observed when comparing T2 vs. T3 (*p <* 0.05) and T2 vs. T5 (*p <* 0.01) in all participants that were determined by using BDI-II (Figure 4C).

Figure 4D–F show the pattern of MRSs in the morning, afternoon and evening for each gender. Significant differences were observed in the anxiety level in males when comparing T1 and T3 (*p* < 0.05; Figure 4D) and depression level when comparing T2 and T3 (*p* < 0.01; Figure 4F) that were determined by using BDI-II. There was no significant difference observed comparing data between male and female concerning MRSs (Figure 4D–F).

Table 5 shows Cohen’s (d) ES of MRSs that computed between the T1 and T3. The ES concerning HADS-A was medium in all participants, large in males only and small in females only. The ES of HADS-D was small in all participants, in males only and females only. Meanwhile, the ES of BDI-II was at a medium level in all participants, in males only and females only.

### 3.5. BC during RF

#### 3.5.1. All Participants

**Figure 5:**
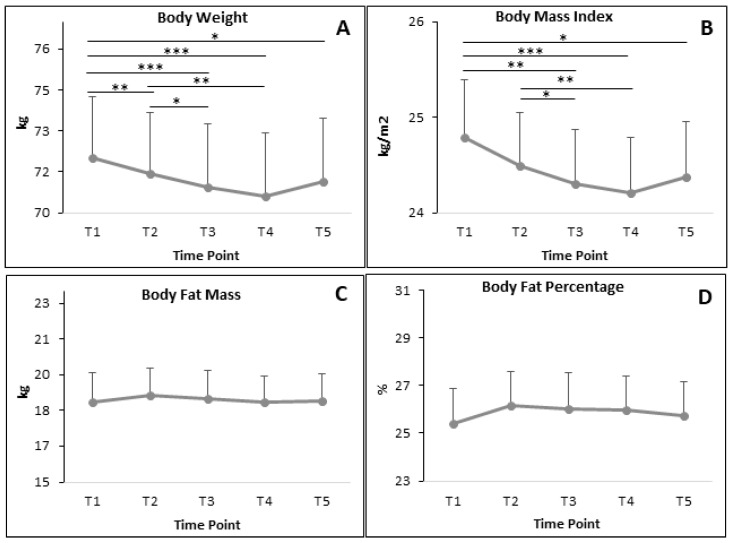

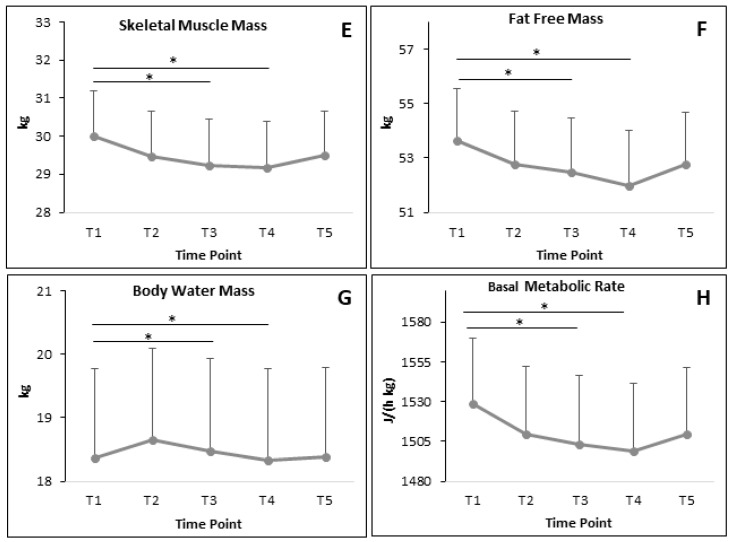
The alteration of BC parameters during RF in all participants (**A**: body weight; **B**: body mass index; **C**: body fat mass; **D**: body fat percentage; **E**: skeletal muscle mass; **F**: fat free mass; **G**: body water mass; **H**: basal metabolic rate). Data presented in mean and SEM. * *p* < 0.05; ** p < 0.01; *** *p* < 0.001.

BC parameters of all participants are demonstrated in Figure 5. BW and BMI significantly decreased almost at every time point (Figure 5A,B) when compared to the baseline T1. It also showed a significant decrease when compared T2 and T3 in both BW and BMI. The significant differences in SMM, FFM, BWM and BMR (Figure 5E–H) were only observed when comparing T1 and T3; and T1 and T4. Meanwhile, there was no significant difference in BFM and BFP (Figure 5C,D).

Table 6 demonstrates the ES of BC parameters of all participants between T1 and T3. The ES of all BC parameters in all participants were low (Table 6).

#### 3.5.2. BC Parameters in Subgroup of Male and Female during RF

**Figure 6:**
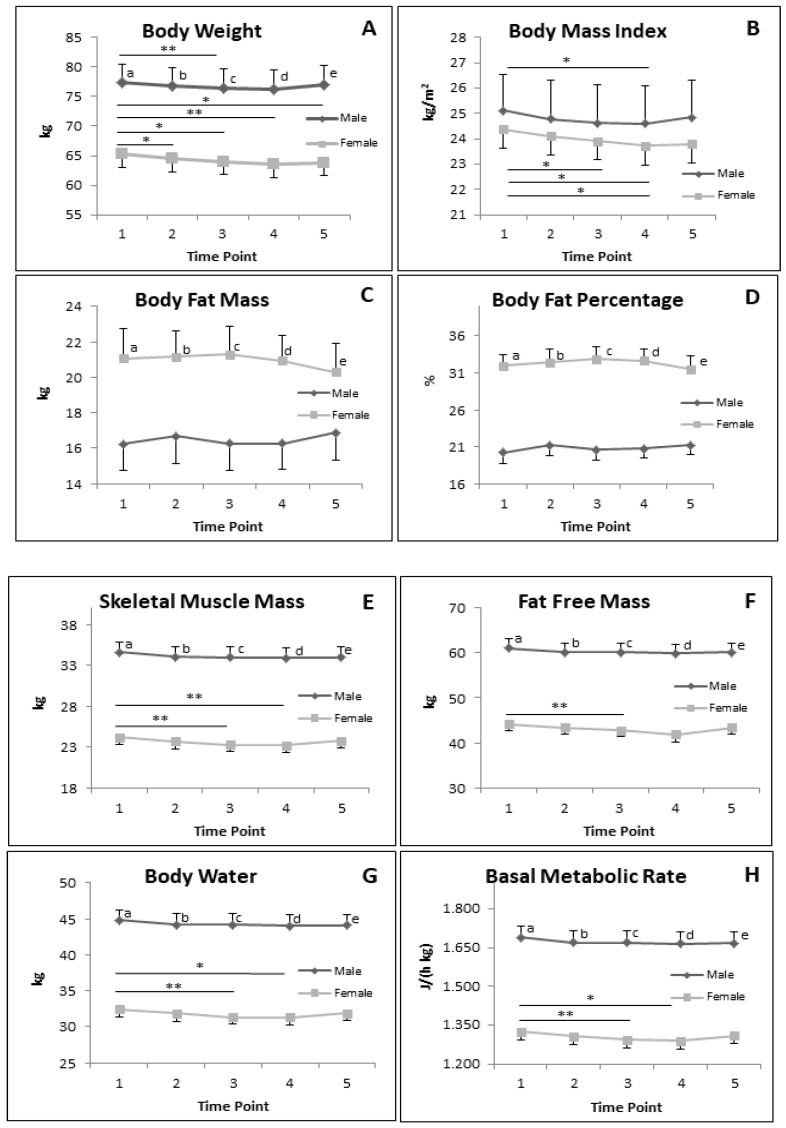
Alteration of BC during RF in subgroup male (*n* = 19) of and female (*n* = 15). (a–e) significant difference between male and female at T1, T2, T3, T4 and T5, respectively. (**A**: Body weight; **B**: Body mass index; **C**: Body fat mass; **D**: Body fat percentage; **E**: Skeletal muscle mass; **F**: Fat free mass; **G**: Body water mass; **H**: Basal metabolic rate). Data presented in mean and SEM. * *p* < 0.05; ** *p* < 0.01.

Figure 6 demonstrates the results of different BC parameters in males and females. In males, significant reductions were only observed in BW and BMI, particularly when comparing T1 and T3; and T1 and T4, respectively. In females, BW and BMI significantly decreased when comparing T1 with all other time points. A similar pattern of significant differences was also observed in BMI of females, except when comparing T1 and T5. BFM and BFP were not significantly different in both males and females along the study.

The significant decrease of SMM, FFM, BWM and BMR were only observed in females. The SMM, BWM and BMR were significant differences when comparing T1 and T3 and T1 and T4 (Figure 6E,G,H). The FFM of female significantly decreases when comparing T1 and T3 (Figure 6F).

All these body composition parameters, except BMI, were significantly different when comparing male and female at T1, T2, T3, T4 and T5.

Table 7 shows the ES of all BC parameters between T1 and T3. The ES of males and females of all BC parameters computed from T1 and T3 were low.

## 4. Discussion

The main objectives of this study wat to determine the effect of RF on fatigue in young male and female participants. Additionally, sleepiness, MRSs and BC parameters were also determined. It was performed in Germany in 2017 where RF was conducted for about 17–18 h/day, due to the summer time.

### 4.1. Fatigue and Sleepiness

As aforementioned, fatigue is one of important parameters that one should be control in order to have good performance in conducting daily activities. As prolonged fasting, like RF, could also affect the performance of daily activities, it is of important to determine the level of fatigue in different time points (morning, afternoon, evening) during RF. Additionally, the sleepiness score was also measured at similar time points.

Significant alterations of fatigue in all participants (the combination of male and female) were observed in the morning and evening time, but not in the afternoon. The alterations were positive—i.e., resulted in a decrease of fatigue. The pattern of alterations of fatigue is quite interesting, the afternoon and evening are similar and tend to decrease until T5. Meanwhile, the morning pattern tends to decrease until T4 and turns back to the baseline level at T5. The resulting study was in agreement with a previous study that showed the improvement of fatigue score in male participants that were measured in the morning [16]. The latter study was only measured until one week after RF (T4). Therefore, our results added new information that the effect of RF on fatigue could only reach until one week after RF. It could be that participants already back to their usual life style one week after RF. This study shows contradictory than the other studies that was done in nurse [19] and elite male judo athletes [30]. In the two latter studies, fatigue levels were increased. It could be that characteristic and activities of participants were different. Another reason could be that the different of assessment tools. Piper fatigue scale and fatigue abbreviated questionnaire were used in the two latter study, respectively [19,30] and FSS in this study.

Sleepiness score was determined by using ESS, which is a validated instrument to evaluate the likelihood that participants will fall asleep during certain activities [28]. The sleepiness score of all participants and also in the only males as well as in the only females in this study was not significantly different. This was in agreement with Bahammam et al. [31] that showed no effect on ESS, but in contrast with the previous study that showed gradual improvement [16]. One of the reasons could be that the latter study studied only male participants; and in the current study, there were no significant differences in male participants only. The sample size could be another reason this study contrasted with the previously referenced study. The current study had about 24% less participants (male only) than compared to the previous study [16].

### 4.2. Mood-Related Symptoms

MRSs were determined by using HADS-A for anxiety, HADS-D and BDI-II for depression. There was a tendency of improvement of the anxiety score of all participants—in males as well as females. However, the significant difference was only observed in male participants. This is consistent with the previous study that showed significant improvement in male participants [16].

The significant improvement of depression score was observed in all participants and in the only males, but not in the only females. This result is consistent with previous studies (References that showed significant improvement in male participants [16,32] and in only BDI-II as an assessment tool, but not in HADS-D [16]. It is known that BDI-II is more sensitive to the alteration of depression in cross-cultural studies [33]. Our results also show that the effect of RF on depression score could be lasted until one month after RF. Improvement of mood which was measured by using BDI-II was also observed in 2-year study of calorie restriction [34].

### 4.3. BC Parameters

BW and BMI of all participants were significantly reduced at all times when compared to before RF (T1). The effect could also still be observed until one month after RF (T5). The results of this study are consistent with other studies [16,35,36,37]. In spite of contradictory to other studies that could not show significant differences during RF [38,39].

Concerning SMM and FFM, it seems not only 18 to 19 h of fasting, but also from 17 h of fasting could start to reduce the SMM significantly. Fasting could increase the gluconeogenesis that leads to protein breakdown [40,41]. However, this result was interesting, as the significant reduction of SMM, FFM and BWM could only be observed in female participants, not in male participants. A previous study reported the reduction of muscle mass in males was observed in fasting for about 18–19 h [16,35]. Some studies agree that there are no differences in protein metabolism of adult men and women [42,43]. However, in certain conditions, like prolonged fasting, it seems the gluconeogenesis with proteolysis of protein is faster in females than males, as it is shown in this study. Further study needs to be performed to elucidate this mechanism.

Subgrouping analysis based on gender, the significant reduction of SMM, FFM, BW and BMR were only observed in females, not in male participants. This is an agreement with Bahammam et al. [44] that showed a reduction of female resting metabolism rate during fasting day as compare to non-fasting day.

### 4.4. Effect Size

The ESs of fatigue, sleepiness, MRSs and BC parameters were only computed between T1 and T3, as it was supposed that T3 (last days of RF) could be the most interesting point that could lead to the highest effect during RF. Our results showed that medium to large effects were observed particularly for MRSs, particularly BDI-II and HADS-A, respectively. These also could lead to the conclusion that the benefit of RF particularly could be seen in the MRSs.

To the best of our knowledge, this is the first study that reported the effect of fatigue in males and females during RF in three different times: morning, afternoon and evening. This is also the first study that reported, medium and large ES of long fasting period (17 h/day) on MRSs.

Limitations of this study. In this study, there was no control group (non-fasting group) to be compared with the fasting group. It was decided as in our previous study there were no group differences between fasting and non-fasting groups in all the above-mentioned parameters [16]. However, in the future it would be of interest to perform another study by comparing it with the non-fasting group with both genders. Another limitation is related to the age group of participants who are mostly young: almost all are university students. In this study, the activities of participants and other circumstances (e.g., daily activities, sport and exam periods) were not recorded. It would be relevant and of importance to, in the future, take into accounts these parameters. Considering the effect of RF could be different in different age groups, future studies should consider recruiting older participants, too. The food intake was not monitored, as we would like to observe the real-life situation of participants during RF. It would be important to monitor food intake, as dietary diversity and food pattern could be different during RF [45,46].

## 5. Conclusions

In summary, engaging in RF could result in various benefits to young males and females, including a decrease in fatigue and improvement of MRSs.

## Figures and Tables

**Table 1:** Study design of RF.

	Time Point of Assessment and Assessment Parameters				
	T1 (Baseline: 1 Week before RF)	T2 (Mid of RF)	T3 (Last Days of RF)	T4 (1 Week after RF)	T5 (1 Month after RF)
**Morning** **07:00–10:00)**	BC, FSS, ESS, HADS, BDI-II	BC, FSS, ESS, HADS, BDI-II	BC, FSS, ESS, HADS, BDI-II	BC, FSS, ESS, HADS, BDI-II	BC, FSS, ESS, HADS, BDI-II
**Afternoon** **(13:00–15:00)**	FSS, ESS	FSS, ESS	FSS, ESS	FSS, ESS	FSS, ESS
**Evening** **(19:00–20:00)**	FSS, ESS	FSS, ESS	FSS, ESS	FSS, ESS	FSS, ESS

Note: RF—Ramadan fasting; BC—body composition; FSS—fatigue severity scale; ESS—Epworth sleepiness scales; HADS—hospital anxiety depression scale; BDI-II—Beck’s depression inventory-II.

**Table 2:** Baseline clinical characteristics of participants.

	All Participants *N* = 34	Male *N* = 19	Female *N* = 15	*p* (Male vs. Female)
Age	25.1 ± 0.8	24.8 ± 1.0	25.5 ± 1.2	0.65
Caucasian/Other	23/11	14/5	9/6	0.48
**Body Composition**				
BW (kg)	72.0 ± 2.3	77.31 ± 3.1	65.3 ± 2.3	<0.01
BMI (kg/m^2^)	24.8 ± 0.6	25.10 ± 0.9	24.4 ± 0.8	0.56
SMM (kg)	30.0 ± 1.2	34.60 ± 1.2	24.2 ± 0.8	<0.001
BFM (kg)	18.4 ± 1.2	16.2 ± 1.7	21.1 ± 1.5	<0.05
FFM (kg)	53.6 ± 1.9	61.1 ± 2.0	44.2 ± 1.4	<0.001
BFP (%)	25.4 ± 1.5	20.2 ± 1.5	31.9 ± 1.6	<0.001
BWM (kg)	39.3 ± 1.4	44.8 ± 1.4	32.4 ± 1.0	<0.001
BMR (J/(h kg))	1528.5 ± 41.4	1686.0 ± 85.3	1324.7 ± 30.3	<0.001
**Mood, Fatigue, Sleepiness**				
Anxiety (HADS-A)	6.2 ± 0.6	6.6 ± 0.6	5.9 ± 1.1	0.57
Depression (HADS-D)	4.4 ± 0.6	4.8 ± 0.7	4.0 ± 0.8	0.49
Depression (BDI-II)	10.9 ± 1.3	9.5 ± 1.3	12.7 ± 2.3	0.21
Fatigue severity scale (Median (IQR))	3.1 (2.1–4.5)	3.0 (2.4–4.4)	3.3 (2.1–4.8)	0.73
Epworth sleepiness scale (Median (IQR))	7.0 (5.0–10.0)	7.0 (5.0–10.0)	7.4 (5.0–10.0)	0.89

Baseline data of age, race, BC parameters, mood, fatigue and sleepiness of all participants, only males and females. *p*-values were tested by using t-test or Mann–Whitney-U test (FSS and ESS).

**Table 3:** Fatigue and sleepiness scores during RF (all participants).

	Time Point					*p*	
	T1 (*n* = 34)	T2 (*n* = 34)	T3 (*n* = 34)	T4 (*n* = 34)	T5 (*n* = 34)		
**Fatigue severity scale; median (IQR)**							
Morning	3.1 (2.1–4.5)	2.6 (2.2–3.9)	2.67 (1.4–3.9)	2.1 (1.2–3.9)	3.0 (1.2–4.2)	<0.05	<0.001 ^§^
Afternoon	3.2 (1.9–4.4)	2.6 (1.8–3.7)	3.15 (1.6–4.7)	2.4 (1.2–3.8)	2.1 (1.2–4.0)	0.16	
Evening	3.4 (2.1–4.6)	2.7 (1.7–4.4)	3.28 (1.5–4.6)	2.5 (1.3–4.1)	2.2 (1.2–3.6)	<0.01	
**Sleepiness (ESS score); median (IQR)**							
Morning	7.0 (5.0–10.0)	7.0 (3.75–10.00)	6.0 (2.0–9.0)	6.0 (1.8–9.2)	6.8 (2.0–10.2)	0.37	0.66 ^§^
Afternoon	7.5 (2.8–10.0)	6.00 (1.8–9.2)	7.50 (3.0–11.2)	8.0 (2.0–11.0)	6.8 (2.0–10.2)	0.44	
Evening	9.0 (5.0–11.0)	7.0 (3.8–10.0)	9.00 (3.0–13.2)	8.5 (4.5–11.2)	7.3 (3.8–11.2)	0.87	

Note: ^§^ Friedman-rank test followed by post hoc-test with Bonferroni correction.

**Table 4:** ES of fatigue and sleepiness scores during RF in the morning, afternoon and evening between T1 and T3.

		ES		
		All Participants	Male	Female
FSS	Morning	0.07	0.08	0.08
	Afternoon	0.01	0.03	0.26
	Evening	0.04	0.00	0.23
ESS	Morning	0.07	0.08	0.01
	Afternoon	0.02	0.00	0.10
	Evening	0.00	0.00	0.05

Note: ES—effect size; FSS—fatigue severity scale; ESS—Epworth sleepiness scale.

**Table 5:** Cohen’s ES of MRSs between T1 and T3.

	ES Cohen’s (d)		
	All Participants	Male	Female
HADS-A	0.54	0.80	0.31
HADS-D	0.29	0.18	0.43
BDI-II	0.54	0.58	0.54

Note: ES: effect size; HADS-A/D—hospital and anxiety depression scale-anxiety/depression; BDI-II—Beck’s depression inventory.

**Table 6:** Cohen’s ES (d) of BC parameters of all participants between T1 and T3.

	ES Cohen’s (d)
BW	0.08
BMI	0.14
SMM	0.11
BFM	0.02
FFM	0.10
BFP	0.07
BWM	0.10
BMR	0.10

Note: ES—effect size; BMI—body mass index; BFP—body fat percentage; BW—body weight; BWM—body water mass; SMM—skeletal muscle mass; BFM—body fat mass; FFM—fat-free mass; BMR—basal metabolic rate.

**Table 7:** Cohen’s (d) ES of BC parameters between T1 and T3.

	ES Cohen’s d	
	Male	Female
BW	0.06	0.14
BMI	0.13	0.16
SMM	0.12	0.30
BFM	0.00	0.04
FFM	0.10	0.27
BFP	0.05	0.15
BWM	0.10	0.27
BMR	0.10	0.27

Note: ES—effect size; BMI—body mass index; BFP—body fat percentage; BW—body weight; BWM—body water mass; SMM—skeletal muscle mass; BFM—body fat mass; FFM—fat-free mass; BMR—basal metabolic rate.
